# Arthroscopic Management of Patellar Instability in Skeletally Immature Patients: Current Concepts and Future Directions

**DOI:** 10.3390/jcm14197085

**Published:** 2025-10-07

**Authors:** Alexandria Mallinos, Kerwyn Jones

**Affiliations:** 1 Rebecca D. Considine Research Institute, Akron Children’s Hospital, Akron, OH 44302, USA; 2 Department of Orthopedic Surgery, Akron Children’s Hospital, Akron, OH 44302, USA

**Keywords:** patellar instability, pediatric, arthroscopy, medial patellofemoral ligament reconstruction, growth-sparing surgery, patellar dislocation, skeletally immature, review

## Abstract

**Background/Objectives**: Patellar instability is a common orthopedic condition affecting pediatric and adolescent populations, particularly during periods of rapid growth and increased sports participation. Recurrent patellar dislocation in skeletally immature patients is frequently associated with underlying anatomical risk factors such as patella alta, trochlear dysplasia, or increased tibial tubercle–trochlear groove distance. **Methods**: This narrative review summarizes the current evidence on the epidemiology, diagnostic approach, and arthroscopic management of patellar instability in skeletally immature patients. **Results**: Arthroscopy has become an essential tool in both the diagnosis and treatment of patellar instability, allowing for minimally invasive assessment of patellofemoral alignment, chondral pathology, and ligament integrity. It also enables precise surgical interventions such as physeal-sparing medial patellofemoral ligament reconstruction, which remains the preferred stabilization technique for patients with open physes due to its safety and efficacy. Emerging innovations, including robotic-assisted tunnel placement, bioengineered scaffolds for cartilage repair, and three-dimensional modeling for surgical planning, have the potential to improve outcomes and arthroscopic surgical precision in this population. Despite these advances, major challenges such as a lack of pediatric-specific outcome measures, variability in surgical indications and rehabilitation protocols, and limited long-term follow-up data remain. **Conclusions**: Optimizing outcomes in pediatric and adolescent patients with patellar instability requires individualized growth-aware strategies and multidisciplinary collaborations. By integrating technological innovation with patient-centered care, clinicians can continue to refine the arthroscopic management of patellofemoral instability in young patients.

## 1. Introduction

Patellar instability is a common cause of knee dysfunction in pediatric and adolescent populations. In the United States, the incidence of patellar dislocation is 31 per 100,000 individuals between the ages of 10 and 19 years [1]. This condition has a substantial impact on the patient’s function, activity level, and quality of life. Patellar instability has been associated with high levels of recurrence rates, especially among skeletally immature individuals where underlying anatomical risk factors, such as patella alta, troclear dysplasia, generalized ligament laxity, and increased tibial tubercle to trochlear groove (TT-TG) distance, have been observed [2,3,4,5,6,7].

Although non-operative management remains the standard of care after a first-time dislocation, especially when there are no high-risk anatomical factors or osteochondral injuries, many patients will continue to experience recurrent instability that requires surgical intervention [8].

Arthroscopy has advanced the diagnosis and treatment of common intra-articular pathologies that frequently accompany patellar dislocation such as loose bodies and chondral lesions [9]. Arthroscopic techniques have enabled minimally invasive approaches for cartilage preservation, the evaluation of patellar tracking, and medial patellofemoral ligament (MPFL) reconstruction [10,11].

In skeletally immature patients, careful surgical planning is required to avoid physeal damage during surgery. Techniques such as growth-aware graft fixation and physeal-sparing MPFL reconstruction have been increasingly utilized in order to protect the open growth plate [12,13,14]. However, despite these efforts, variations in surgical techniques and outcome reporting remain a challenge due to a lack of high-quality, long-term, pediatric-specific data. In addition, rehabilitation protocols customized to patients with open physes are vital to restore function while protecting surgical outcomes in this population.

Recent innovations, such as patient-specific instrumentation, robotic-assisted arthroscopy, and three-dimensional (3D) preoperative planning, have the potential to improve surgical accuracy and outcomes [14,15,16,17,18]. Additionally, biological augmentation using tissue scaffolds or orthobiologics may offer alternative strategies to enhance healing in instances where cartilage damage or recurrent instability are present [19]. When integrated with established arthroscopic techniques, these technologies have the potential to further improve care and surgical outcomes for patients with patellar instability.

The purposes of this narrative review were to summarize current clinical evidence, surgical advancements, and pediatric-specific considerations for arthroscopic management of patellar instability and to inform best practices, support individualized care strategies, and guide future research to improve outcomes in skeletally immature patients with patellar instability.

## 2. Methods

This narrative review was conducted using a structured search and appraisal approach to ensure comprehensive coverage of the literature. PubMed and Google Scholar databases were queried for studies published between January 2000 and June 2025. English language search terms consisted of: “patellar instability”, “adolescent”, “skeletally immature”, “pediatric”, “arthroscopy”, “arthroscopic management”, “medial patellofemoral ligament reconstruction”, and “growth-sparing techniques”.

Eligible studies included patients younger than 18 years of age with patellar instability who underwent arthroscopic or arthroscopy-assisted procedures. Inclusion was limited to articles reporting clinical outcomes, technical innovations, and diagnostic approaches. Studies restricted to skeletally mature populations, editorials, single case studies, and expert opinion without clinical data were excluded.

Evidence quality was appraised according to the Oxford Centre of Evidence-Based Medicine (OCEBM) criteria, with most key pediatric patellar instability studies representing Level III (retrospective cohort) or Level IV (case series) evidence. Fewer prospective studies (Level II) were obtained and no randomized controlled trials specific to arthroscopic physeal-sparing techniques were utilized. Thus, this relative strength of evidence was considered when summarizing treatment recommendations and knowledge gaps.

## 3. Epidemiology and Etiology of Patellar Instability in Skeletally Immature Patients

Patellar instability is most common in individuals between 10 and 17 years of age, with the highest incidence reported in early adolescence [20]. Females have a higher incidence of patellar instability than males [21]. The age of first-time patellar dislocation coincides with periods of increased participation in sports and rapid skeletal growth [2,20]. Nearly 70% of initial patellar dislocations for this patient demographic occur due to external trauma caused by a sporting activity [22]. Recurrence rates after nonoperative management can range from 30 to 70%, with a higher likelihood of occurring if underlying anatomical abnormalities are present [23].

There are several biomechanical and anatomical factors that contribute to the development of patellar instability in pediatric and adolescent patients. Some of these risk factors include patella alta, trochlear dysplasia, increased TT-TG and tibial tubercle-posterior cruciate ligament (TT-PCL) distance, ligament laxity, and femoral anteversion [2,3,4,5,6,7]. Trochlear dysplasia, in particular, has been found in up to 85% of patients with recurrent instability [24]. In skeletally immature populations, a larger TT-TG distance is associated with lateralization of the extensor mechanism, which further contributes to patellar instability [25]. Analogous to TT-TG, elevated TT-PCL values suggest significant lateral and rotational malignment [26,27,28,29]. In instances where patella alta is present, the risk of lateral subluxation is increased as the bony constraint offered by the trochlear groove during knee flexion is reduced [14].

These risks factors are further complicated by the presence of open growth plates in skeletally immature patients, thus influencing surgical decision-making and constraining certain interventions. Additionally, the presence of neuromuscular conditions, such as cerebral palsy or ligamentous laxity syndromes, may increase the likelihood of recurrent dislocation, further complicating standard treatment approaches [7,30]. Classification and recognition of these anatomical and biomechanical risk factors are essential to determine the appropriate course of care and treatment, ultimately resulting in tailored, patient-specific interventions to the pediatric population. Emerging imaging techniques, such as weight-bearing magnetic resonance imaging (MRI) [31] and 3D computational modeling [32] may enhance the precision of preoperative planning and provide additional insight into complex anatomic contributors to instability.

## 4. Diagnostic Approach to Patellar Instability

### 4.1. Clinical Evaluation

Accurate diagnosis of patellar instability in pediatric and adolescent populations requires a combination of thorough clinical evaluation and advanced imaging techniques. Clinical history should document the mechanism of injury, the number of prior subluxation events, and functional limitations during movement. Physical examination often reveals tenderness along the MPFL, a positive apprehension test, lateral patellar hypermobility, and an increased passive lateral translation of the patella during early flexion angles [33,34].

### 4.2. Radiographic Evaluation

**Figure 1:**
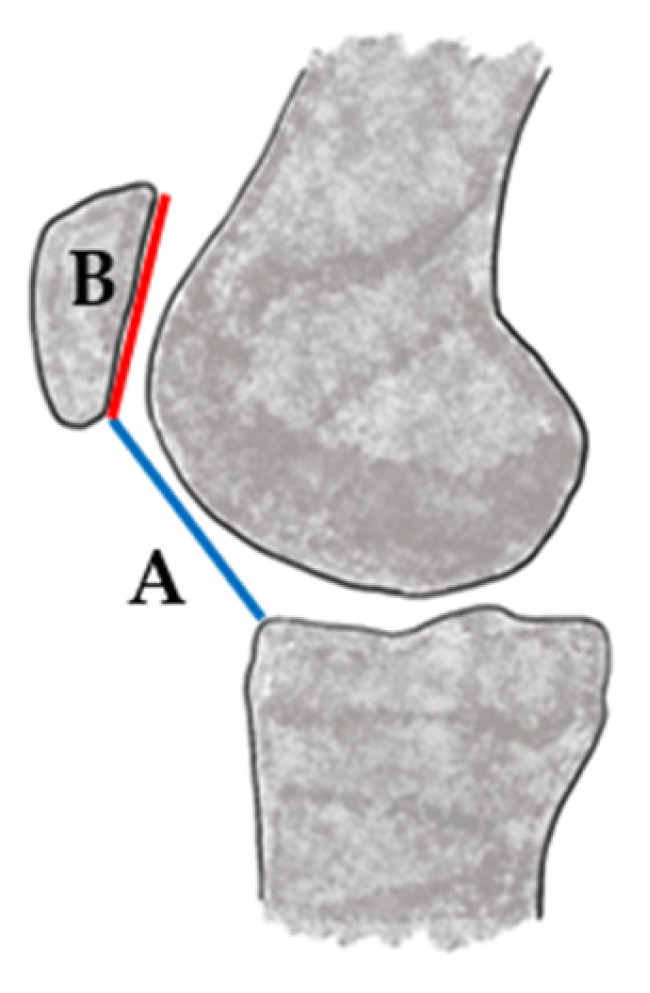
Caton–Deschamps index (A/B): obtained by dividing the distance from the inferior pole of the patella to the tibial plateau (A—blue) by the length of the patellar articular surface (B—red).

Imaging plays a critical role in confirming the diagnosis and identifying any anatomical risk factors that may contribute to patellar instability (Table 1). Standard radiographs should include anteroposterior (AP), lateral, axial (sunrise or Merchant) views, as well as views that evaluate trochlear morphology, patellar height, and determine if there is evidence of osteochondral injury [35]. Patella alta is frequently quantified using the Caton–Deschamps index (or the Insall–Salvati ratio in skeletally mature populations) with values greater than 1.2 suggestive of abnormal patellar positioning [36,37] (Figure 1).

### 4.3. Updated Dejour Classification and the Menu à la Carte

The Dejour classification system has historically been used to categorize trochlear dysplasia into types A through D based on lateral radiographic findings such as the presence of double contours, supratrochlear spurs, and a crossing sign [41,42]. However, recently Dejour and colleagues introduced an updated classification that redefines trochlear dysplasia by integrating radiographic and MRI features with functional assessment of patellofemoral tracking [40]. This revised framework emphasizes dynamic evaluation and incorporates new MRI-based parameters that enhance diagnostic precision and risk stratification (Table 2).

This revised framework links imaging findings with the updated *menu à la carte* that associates specific dysplasia patterns with tailored treatment options offering a more precise basis for risk stratification and surgical decision-making, particularly regarding the indications for tracheoplasty.

The concept of the *menu à la carte* has been central to guiding surgical decision-making from patellofemoral instability since it was first introduced by Dejour and colleagues in the 1990s, when the original classification of trochlear dysplasia was paired with corresponding surgical procedures to address distinct anatomic abnormalities [41]. During the 2000s and 2010s, the framework evolved to incorporate emerging procedures, most notably MPFL reconstruction and TTO, reflecting increasing recognition of the role of the soft tissue stabilization and extensor mechanism realignment in restoring patellar stability [41,43,44]. In 2021, Dejour and colleagues published an updated version of the *menu à la carte*, expanding the algorithm to integrate multimodal imaging and clarifying the indications for trochleoplasty relative to soft tissue procedures [45].

The most recent update in 2025 [40] represents a major advance, as it introduced both a redefined Dejour classification of trochlear dysplasia and a revised *menu à la carte* that explicitly links diagnostic parameters with individualized treatment pathways. Unlike earlier iterations, which relied heavily on static radiographic morphology, the updated classification incorporates MRI-based assessment, functional evaluation of patellofemoral tracking, and dynamic risk stratification.

The decision logic now emphasizes combining or sequencing procedures such as MPFL reconstruction, soft tissue balancing, realignment osteotomies, and tracheoplasty based on a comprehensive appraisal of dysplasia severity, patient-specific factors, and alignment abnormalities. This historical progression underscores the transformation of the *menu à la carte* from a descriptive classification tool into a dynamic, clinically integrated framework that continues to shape the management of patellofemoral instability worldwide.

### 4.4. MRI-Based Exploration

MRI provides a detailed evaluation of the MPFL, articular cartilage, and bone bruising patterns consistent with recent subluxation events [5,46,47,48]. In addition, MRI can be used to measure TT-TG and TT-PCL distance. Measurements greater than 20 mm are considered pathologic in most patients [25,29]. However, in skeletally immature populations, values between 15 and 20 mm may fall within a borderline range [39] and should be interpreted within the context of skeletal maturity, trochlear morphology, patellar height, and the presence of clinical instability. Because anatomical variation is more pronounced in growing individuals, the TT-TG distance should be considered alongside other risk factors rather than as an isolated indicator for surgical intervention. MRI is also useful to identify osteochondral fractures or loose bodies that may necessitate arthroscopic intervention [9]. MRI should be obtained when a loose body of any size is seen on plain radiographs as even a small loose body may involve a rather large articular component that is more accurately evaluated on MRI.

MRI critically allows visualization of the distal femoral and proximal tibial physes, informing growth-sparing surgical strategies. Assessment of the growth plates is essential when evaluating and managing patellar instability in skeletally immature patients. Surgical planning must account for the location and status of the physes to avoid iatrogenic growth disturbances which can lead to angular deformities or limb-length discrepancies [49]. MRI is the preferred imaging modality for physeal evaluation because it provides high-resolution visualization of cartilaginous structures and ossification centers without radiation exposure. In the context of MPFL reconstruction, identification of the femoral physis is critical in preventing tunnel placement that compromises the growth plate. Techniques that place the femoral graft fixation distal to the physis or utilize epiphyseal or physeal-sparing constructions are recommended on skeletally immature patients [50]. Accurate physeal assessment also helps stratify patients into growth-based treatment classifications and allows for the determination of timing surgical intervention. Ultimately, incorporating growth plate evaluation into the diagnostic process supports safe, individualized care that balances the need for stabilization with the preservation of future growth potential.

### 4.5. Advanced and Emerging Imaging Modalities

Emerging technologies have enhanced diagnostic precision. Weight-bearing and dynamic MRI techniques offer improved evaluation of patellofemoral tacking and joint congruity under physiologic conditions, although they are currently not widely used in pediatric settings [31,51,52]. Similarly, 3D reconstruction from MR and CT imaging can provide accurate assessments of trochlear morphology in patients with patellar instability [53,54]. Functional video-based assessments and gait analysis may complement structural imaging in complex or recurrent cases by evaluating dynamic malalignment [55,56].

### 4.6. Diagnostic Arthroscopy

While non-invasive imaging remains the standard for initial assessment, diagnostic arthroscopy serves as a valuable adjunct in select cases, particularly when symptoms or imaging findings are inconclusive. Arthroscopy enables direct visualization of the articular cartilage, MPFL attachment sites, and the patellofemoral articulation under dynamic conditions, allowing for real-time assessment of patellar tracking and engagement within the trochlear groove [57]. This is especially advantageous in individuals with mechanical symptoms, such as catching, persistent pain, locking, and when chondral injuries may be suspected but inconclusive on MRI. Arthroscopy can also aid in confirming intra-articular lesions, characterizing cartilage damage severity [58,59], thus guiding treatment decisions, such as loose body removal, cartilage debridement, or early surgical stabilization [59,60]. In the context of recurrent instability, arthroscopy can also be combined with definitive procedures such as MPFL reconstruction or lateral release, providing both diagnostic and therapeutic value [59]. Although not routinely used as a first-line tool, arthroscopy remains an important diagnostic option, particularly in complex, symptomatic, or high-demand pediatric and adolescent patients.

### 4.7. Indications for Surgical Intervention and Decision-Making

Indications of surgical intervention typically include first-time dislocations accompanied by substantial loose bodies and recurrent dislocations that markedly impact functionality and quality of life [9,61]. A first-time patellar dislocation should be treated conservatively unless it is accompanied by substantial loose bodies, which may necessitate immediate surgical intervention to prevent further cartilage damage [62]. Recurrent dislocations significantly impair knee function and quality of life, often requiring surgical stabilization, particularly when conservative management has failed to prevent recurrent instability [63].

An algorithmic approach for surgical decision-making involves evaluating several key factors. Patient history is crucial. Differentiating traumatic versus atraumatic dislocations guides urgency and the appropriate surgical technique. Traumatic dislocations typically result from direct injuries or major external forces, often necessitating immediate intervention to address acute damage, such as osteochondral fractures or large loose bodies. Conversely, atraumatic dislocations are usually associated with underlying anatomical or ligamentous deficiencies and recurrent instability, prompting a thorough assessment of alignment and ligamentous integrity to guide elective surgical stabilization.

Alignment assessment across multiple planes is necessary. Coronal plane alignment identifies conditions such as genu valgum or varum. Axial plane analysis involves TT-TG and TT-PCL distances, highlighting lateralization and rotational abnormalities. The sagittal plane assessment evaluates patellar height and patellar tilt. Additionally measuring generalized ligamentous laxity informs whether soft tissue procedures or additional stabilization might be required [64].

## 5. Surgical Techniques

Arthroscopy plays a vital role in the surgical management of recurrent patellar instability. Minimally invasive, arthroscopic surgical interventions provide visualization, access, and treatment for patellofemoral pathology. In pediatric populations, arthroscopic techniques must be adapted to accommodate open physes and variable skeletal maturity. While physeal-sparing MPFL reconstruction remains the gold-standard surgical intervention in pediatric populations, additional procedures such as tibial tubercle osteotomy (TTO) or trochleoplasty may be contraindicated for skeletally immature patients with chronic instability or severe dysplasia.

**Figure 2:**
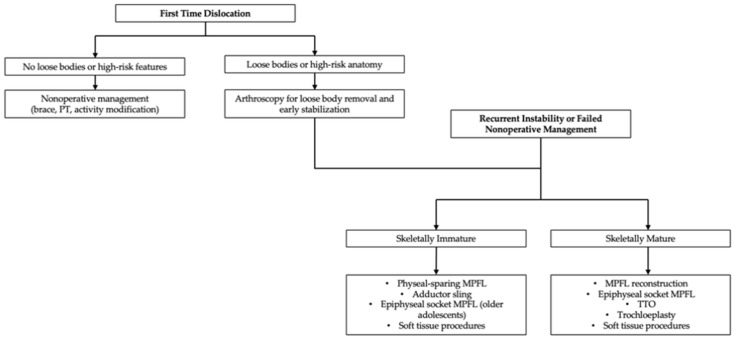
Treatment pathway for patellar instability management.

Soft tissue arthroscopic procedures, including lateral release or imbrication, may be performed arthroscopically to address abnormal patellar tracking and reduce lateral patellar tilt. These techniques adjust the tension in surrounding soft tissues to enhance stability. Conversely, bony procedures like TTO involve repositioning of the tibial tubercle to correct biomechanical alignment and reduce lateralization of the extensor mechanism. However, due to the proximity of the growth plates, such bony interventions carry risks of physeal injury and resultant growth disturbances and are typically reserved for patients nearing skeletal maturity. Similarly, trochleoplasty, which reshapes the trochlear groove, is contraindicated in younger patients due to the risk of damage to developing cartilage and growth plates. Thus, this section outlines key arthroscopic techniques and their pediatric-specific considerations, emphasizing the delicate balance required to effectively manage patellar instability while preserving growth potential. Building upon these diagnostic considerations, the following treatment pathway (Figure 2) outlines evidence-based management options tailored to skeletal maturity.

### 5.1. Medial Patellafeoral Ligament Reconstruction

MPFL reconstruction is the most commonly performed surgical intervention for recurrent lateral patellar instability. The MPFL serves as the primary constraint to lateral translation of the patella between 0° and 30° of knee flexion, with its insufficiency nearly universal after a first-time subluxation [65]. Reconstruction is indicated in patients with recurrent instability or a first-time dislocation accompanied by the presence of anatomical risk factors, loose bodies, or chondral damage [43].

Graft options for MPFL reconstruction include autografts such as the gracilis or semitendinosus tendon, allograft tissue, or synthetic materials. Most pediatric studies favor allografts due to improved graft survivorship, low cost and revision rate, and clinical outcome scores, and reduced donor-site morbidity [66]. Graft fixation techniques vary and may include interference screws, suture anchors, or cortical buttons [67,68]. Autografts remain acceptable alternatives when tendon quality is sufficient. Pediatric reconstructions generally use a 5–6 mm diameter double graft (18–22 cm length), balancing adequate strength with minimized patellar fracture risk [66].

The femoral tunnel position is critical for restoring isometry and avoiding graft over-constraint as incorrect placement may result in altered patellar tracking or increased contact pressures [67,68]. In skeletally immature patients, physeal-sparing MPFL reconstruction is essential to avoid iatrogenic damage to the distal femoral physis [13]. Given the proximity of the MPFLs anatomical femoral insertion (Schöttle point) to the physis, conventional tunnel-based fixation techniques used in adults pose a significant risk if adapted directly in pediatric patients. To mitigate this risk, a number of surgical strategies have been developed that respect the growth plate while restore patellofemoral stability. Techniques include using an epiphyseal femoral socket, soft tissue fixation (adductor sling), and partial tunnel constructs that avoid transphyseal drilling [13,69,70]. These approaches have shown favorable short-term outcomes with low reinjury rates and minimal complications [13,69,70]. Adductor sling techniques utilize the adductor magnus tendon as a point of graft fixation by looping the graft around it, avoiding the need for bony tunnels altogether. This method has been shown to preserve femoral physis integrity and is particularly suitable for younger children with substantial growth remaining [69,70,71,72]. This technique is relatively straightforward, reproducible, and eliminates the risk of physeal injury. Outcomes have demonstrated re-dislocation rates below 10–15% and favorable return-to-sport rates in short- to mid-term series [69,70,71,72]. However, limitations include the potential for graft lengthening or creep over time due to soft-tissue fixation, which may increase the risk of recurrent laxity [71].

Another approach involves creating a small epiphyseal socket, allowing for anatomical femoral fixation within the epiphysis without violating the physis. This technique is generally suited for older children and adolescents nearing skeletal maturity, when the distal femoral epiphysis provides adequate bone stock for a short tunnel. The socket is typically 4–6 mm in diameter and 15–20 mm in depth [73], drilled entirely under fluoroscopic or navigational guidance to ensure accurate placement and to avoid physeal violation [70]. Reported outcomes are favorable, with re-dislocation rates in the 5–15% range and good functional recovery [70]. Complications, though uncommon, include tunnel malposition with epiphyseal breach, hardware irritation, and graft over-constraint if the femoral socket is placed too proximally or posteriorly.

Some surgeons have also used soft tissue-only constructs which involve anchoring the graft to the medial epicondyle periosteum or employing suture anchors placed in the distal femoral epiphysis [74,75,76]. These methods are particularly useful in very young patients with limited epiphyseal bone stock, where even a short socket may be unsafe. Early outcomes demonstrate re-dislocation rates typically under 20%, with satisfactory scores and pain reduction [77]. However, because fixation depends on soft tissue purchase, complications include anchor pull-out, gradual graft laxity, and higher reoperation rates compared to bony fixation [77]. Despite these limitations, soft-tissue fixation remains a valuable physeal-sparing option in the youngest or most skeletally immature patients.

When it comes to patellar fixation, several options exist; however, strategies that minimize fracture risk are recommended in pediatrics. Two low-profile medial anchors or small unicortical sockets are often favored, as they limit bone removal and allow independent limb tensioning [78,79]. Trans-osseous sutures through short, protected tunnels are another alternative, though they carry greater risk of fracture if oversized or malpositioned [80]. Large full-thickness transverse tunnels, while biomechanically strong, are generally avoided in skeletally immature patients due to high fracture risk [80].

Graft fixation is typically performed at 30–45° of knee flexion, with the patella centered in the trochlear groove to preserve near-isometric behavior [81]. Cadaveric biomechanical data confirms that fixation at full extension (0°) raises medial patellofemoral contact pressures, whereas fixation at 30° or 60° restores both pressure profiles and patellar tracking to near-normal levels [82]. To avoid over-constraint, intraoperative tension is set to allow about one quadrant of lateral translation at 30°, with smooth tracking confirmed at 0°, 30°, and 60° [83]. This technique reduces the risk of over-constraint and excessive contact pressures, which can occur if the graft is fixed in extension or over-tensioned.

Following graft fixation, intraoperative checks should confirm isometry through the arc of motion, with minimal length change from 0 to 60°. Patellar tracking should be smooth without excessive medial tightness or an exaggerated J-sign. Fluoroscopic documentation of femoral tunnel positioning and photographic evidence of patellar tracking is recommended for the operative record. Importantly, routine lateral release is discouraged, as it risks overcorrection and has been associated with high rates of postoperative medial instability [84,85,86,87]. In cases where significant lateral retinacular tethering persists despite appropriate graft reconstruction, a graded lateral lengthening may be selectively considered as a safer adjunct [88]. Technical pitfalls that can compromise outcomes include femoral tunnel malposition (too proximal or transphyseal) [89], graft over-tensioning [90], fixation at inappropriate flexion angles [91], patellar fracture from oversized tunnels [89], and neglecting to verify isometry intraoperatively.

When it comes to skeletally immature patients, MRI-based physeal mapping is recommended preoperatively to assess the location and extent of open growth plates and to tailor the surgical approach accordingly. This imaging also aids in identifying other concurrent anatomical risk factors which may influence graft tensioning and positioning. While long-term outcome data remain limited, the literature has reported re-dislocation rates below 25%, with higher rates of return-to-sports and minimal complication profiles in patients undergoing physeal-sparing reconstruction [69,74,92,93].

As surgical instrumentation and imaging techniques continue to evolve, newer methods such as 3D personalized navigation templates [94] and all-epiphyseal tunnel drilling with navigation [13] may help further improve surgical precisions, reproducibility, and patient outcomes with this demographic. Ultimately, MPFL reconstruction in skeletally immature patients demands a careful balance between growth plate preservation and anatomic restoration. Table 3 summarizes fixation strategies for physeal-sparing MPFL reconstruction in skeletally immature patients, highlighting their indications, advantages, limitations, and level of evidence.

### 5.2. Tibial Tubercle Osteotomy: Caution in Skeletally Immature Patients

TTO is an arthroscopic procedure that is typically reserved for skeletally mature patients with persistent instability and lateralization of the extensor mechanism, in combination with anatomical risk factors such as TT-TG greater than 20 mm and significant patella alta [44]. The goal of TTO is to distalize, medialize, or anteriorize the tibial tubercle to normalize patellar tracking and reduce lateral patellofemoral joint stress [44].

In skeletally immature patients, TTO is contraindicated due to the proximity of the osteotomy site to the tibial tubercle apophysis and the proximal tibial physis [100]. Thus, disruption to the open physes increases the risk of angular deformities, growth disturbances, and physeal arrest [49]. Even with patients in late adolescence, careful assessment of skeletal maturity is essential prior to considering surgical interventions such as TTO to avoid iatrogenic damage to the growth plates. When indicated in skeletally mature adolescents, arthroscopic-assisted planning with radiographic measurements (Caton–Deschamps index, TT-TG, etc.) should help guide the extent and direction of the correction.

One safe method to correct coronal plane malalignment as documented by increased TT-TG or TT-PCL distance is the Roux Goldthwait procedure in which the patellar tendon is split longitudinally in the central portion of the tendon, the lateral half is detached distally and then passed under the medial portion to resecure it with suture stabilization to the periosteum of the proximal tibia just medial to the native medial portion. This can be used in combination with the MPFL reconstruction or in isolation [101].

### 5.3. Trochleoplasty

In recent years, arthroscopic trochleoplasty has emerged as a minimally invasive alternative to traditional open techniques for the correction of high-grade trochlear dysplasia [102]. This technique is indicated for symptomatic patients with Dejour 2025 high-grade dysplasia [40] (types requiring surgical containment procedures) in whom nonoperative care of isolated soft-tissue procedures are insufficient and should only be considered in adolescents or young adults nearing skeletal maturity. It remains contraindicated in skeletally immature patients with open physes, given the risk of physeal injury and long-term growth disturbances.

Among the most widely described methods is the thin-flap arthroscopic trochleoplasty popularized by Capella and colleagues [102]. This technique involves a stepwise approach: (1) diagnostic arthroscopy to evaluate patellofemoral tracking and associated chondral pathology; (2) use of a C-arm (fluoroscopic) system to localize the limits of the dysplastic trochlea; (3) creation of a thin osteochondral flap over the supratrochlear spur using an arthroscopic burr; (4) recession of the subchondral bone to deepen and reshape the trochlear groove; and (5) fixation of the thin flap back into position, ensuring congruent contour and stability.

Clinical outcomes of arthroscopic trochleoplasty have been increasingly reported. Blønd and Barfod [103] demonstrated that arthroscopic recession trochleoplasty, when performed in conjunction with MPFL reconstruction, significantly improved trochlear morphology on post-operative MRI and led to improve patient-reported outcome scores. These findings suggest that arthroscopic approaches can achieve comparable short- to mid-term outcomes relative to open techniques, with potential benefits in terms of cosmesis and recovery.

Compared with open trochleoplasty, the arthroscopic approach is less invasive, preserves the surrounding soft tissues, and allows for concurrent assessment and treatment of intra-articular pathology. Fluoroscopic assistance provides accurate visualization of the bony contour, reducing the risk of over- or under-correction. Patients may benefit from decreased postoperative pain, faster rehabilitation, and fewer wound-related complications.

However, some challenges should be addressed. Arthroscopic trochleoplasty remains technically demanding, with a steep learning curve. Risks include the creation of an unstable flap if subchondral support is insufficient, or conversely inadequate deepening that leaves residual dysplasia. Over correction may lead to excessive trochlear constraint, while under-correction fails to restore stability. Post-operatively, protocols emphasize protected weight-bearing and progressive return-to-sport after 4–6 months, balancing flap healing with functional recovery [104]. Long-term outcome data are limited with most published studies reporting short- to mid-term follow-up [105,106,107]. Additionally, the safety and durability of the thin-flap technique in younger patients with higher activity levels remain to be validated.

Overall, arthroscopic trochleoplasty represents a promising, less invasive alternative to open trochleoplasty for high-grade dysplasia in skeletally mature adolescents and young adults. Future multicenter studies and long-term follow-up are required to determine its role relative to established open procedures.

### 5.4. Soft Tissue Procedures: Lateral Release and Imbrication

Soft tissue procedures, such as lateral release and imbrication, can be performed arthroscopically to address patellar maltracking and instability, particularly when excessive lateral retinacular tightness or patellar tilt is identified. Although typically supplementary rather than primary treatments, these methods can be beneficial in patients with less severe instability or minimal anatomical abnormalities [14,61].

Lateral release involves arthroscopically transecting the lateral retinaculum to reduce lateral tethering forces acting on the patella. This can help correct lateral patellar tilt and mildly improve medial patellar tracking. However, isolated lateral release has been increasingly scrutinized due to its inconsistent outcomes, high recurrence rates, and risk of iatrogenic medial instability if overly aggressive or improperly indicated [72,75]. Despite these limitations, lateral release remains useful when appropriately selected for patients with significant lateral retinacular tightness and minimal underlying anatomical deformities, especially when used in conjunction with MPFL reconstruction [61,75].

Medial imbrication involves tightening or reinforcing the medial retinacular and capsular structures, typically performed in combination with MPFL reconstruction or other medial stabilization techniques. This method aims to enhance medial patellar support by restoring proper medial soft tissue tension. Medial imbrication is advantageous for skeletally immature patients where growth plate preservation restricts bony corrective interventions. When effectively executed, medial imbrication can improve patellar tracking and reduce instability without substantial risk to the physes [14,74].

Careful patient selection, based on detailed anatomical assessments and growth considerations, is crucial to optimize the outcomes of these procedures. Anatomical assessments should include thorough evaluation of patellar height, trochlear groove morphology, alignment of the lower extremity, and rotational abnormalities. In skeletally immature patients, careful consideration of growth plate status and potential implications for future growth and development is paramount. Overly aggressive soft tissue correction can lead to postoperative complications like medial over-constraint, which may result in stiffness or limited patellar mobility, requiring further interventions, or reduced patellar tracking that negatively impacts functional outcomes. Therefore, individualized treatment planning that integrates both preoperative imaging findings and clinical examinations, along with precise arthroscopic evaluation, remain essential for successful implementation of these adjunctive soft tissue techniques [14,61,74]. Collaborative approaches involving pediatric orthopedic specialists, rehabilitation therapists, and imaging specialists can further enhance patient outcomes and minimize the risks associated with these procedures.

A summary of patellar instability procedures can be found in Table 4.

## 6. Innovations and Future Directions

Standard surgical techniques for patellar instability in skeletally immature patients, while effective in many cases, face important limitations that create gaps in clinical application. Conventional MPFL reconstruction, though widely performed, carries risks of physeal injury given the proximity of the femoral insertion into the distal femoral physis. Outcomes may also be compromised by tunnel malposition or graft over-constraint, leading to recurrent instability or altered patellar tracking. Similarly, TTO and trochleoplasty, though effective in skeletally mature patients, are generally contraindicated in younger populations due to the risk of growth disturbances and long-term joint sequelae. Even with physeal-sparing adaptations, soft tissue-only fixation methods may result in graft creep or recurrence, while reliance on two-dimensional imaging can limit the ability to account for complex three-dimensional risk factors such as rotational malalignment. Collectively, these challenges underscore the need for strategies that improve surgical precision, preserve growth potential, and address associated chondral damage.

Advancements in surgical techniques, imaging modalities, and biologic therapies continue to expand treatment options for patellar instability, particularly in skeletally immature patients. Recent innovations, such as robotic-assisted navigation, bioengineered scaffolds, and 3D patient-specific surgical planning, offer promising improvements to enhance tunnel placement accuracy, address the unmet need of concomitant chondral damage, and provide detailed visualization enabling individualized alignment corrections.

Together, these novel approaches (Table 5) hold promise to refine current practices by improving safety in skeletally immature patients, enhancing surgical reproducibility, and offering biologically restorative solutions where standard reconstruction falls short.

### 6.1. Robot-Assisted Navigation

Robot-assisted navigation is an emerging and increasingly utilized orthopedic procedure used to enhance surgical precision. In the context of MPFL reconstruction, robotic systems facilitate accurate tunnel placement, minimizing the risk of iatrogenic physeal damage in skeletally immature patients. By precisely navigating anatomical landmarks and growth plate boundaries, robotic assistance helps avoid inadvertent damage while optimizing graft positioning and tensioning. A study in 2025 confirmed the effectiveness and safety of robot-assisted individualized MPFL reconstruction, demonstrating accurate femoral tunnel placement within the distal epiphysis, reduced operative variability, and favorable early clinical outcomes, underscoring its potential as a standard adjunct in pediatric patellar instability management [18]. Although early outcomes are encouraging, current data remains limited to small single-center retrospective series (e.g., *n* = 20–40 patients, follow-up less than 2 year) [18]. These studies demonstrate proof of concept and improved tunnel accuracy but lack long-term multicenter validation.

### 6.2. Bioengineered Scaffolds for Cartilage Restoration

Bioengineered scaffolds are at the forefront of cartilage repair strategies to restore articular cartilage integrity in patients with recurrent patellar instability. Designed to mimic the extracellular matrix, these scaffolds support chondrocyte proliferation and matrix deposition. Recent advancements include the development of zonal osteochondral scaffolds with bioactive cartilage zones, ultimately resulting in the promotion of endogenous cell recruitment and cartilage regeneration [108]. Such scaffolds can be integrated into arthroscopic procedures offering a minimally invasive solution for cartilage restoration in skeletally immature patients. This technology is particularly advantageous in pediatric orthopedic patients, where preserving joint health and function during crucial development periods is essential for optimal long-term outcomes. However, most published work [108] remains confined to preclinical animal models and early feasibility trials, with very limited pediatric clinical data. Sample sizes are small (less than *n* = 30), and long-term graft integration and durability remain unproven.

### 6.3. 3D Modeling and Patient-Specific Surgical Planning

3D modeling and patient-specific surgical planning have revolutionized the way complex orthopedic conditions are approached, particularly in pediatric patients with patellar instability. Through advanced imaging modalities, 3D reconstructions of the patellofemoral joint can be generated to visualize the bony and cartilaginous anatomy in detail [53,109]. These models allow surgeons to evaluate critical anatomical risk factors more accurately than with traditional two-dimensional (2D) imaging alone.

For arthroscopic procedures, 3D modeling improves preoperative planning and intraoperative navigation, especially when treating high-grade trochlear dysplasia or complex instability patterns. In skeletally immature patients, where preserving open growth plates is essential, 3D models help identify safe zones for tunnel placement during MPFL reconstruction, reducing the risk of iatrogenic physeal damage. By simulating different surgical scenarios such as femoral tunnel trajectories or graft lengths, surgeons can individualize procedures based on the patient’s anatomy resulting in safer and more effective surgical outcomes [110,111,112].

Beyond surgical execution, 3D modeling facilitates better communication with patients and families by offering a tangible visualization of the pathology and planned intervention. This is especially valuable in pediatric populations, where patient and parent understanding and engagement are crucial components of informed consent and postoperative compliance.

As the integration of artificial intelligence and automated segmentation tools advances, the efficiency and accessibility of 3D modeling will continue to improve. The incorporation of these technologies into pediatric knee arthroscopy not only enhances surgical safety and precision, but also represents a step towards personalized, data-driven orthopedic care.

Unfortunately, modeling approaches largely derive from case series and pilot studies (sample sizes typically less than 50), that are retrospective in nature. While 3D modeling improves anatomical understanding and operative planning, robust prospective studies comparing outcomes to standard care are lacking [53,109,110,111,112]. Cost and limited access to advanced imaging and software may also restrict widespread adoption.

## 7. Knowledge Gaps and Research Needs

Despite the growing adoption of arthroscopic techniques for managing patellar instability, important knowledge gaps remain, particularly in skeletally immature populations. Many current surgical workflows and rehabilitation protocols are derived from adult studies, with limited pediatric-specific data available to guide individualized care. Addressing these deficiencies is essential for optimizing outcomes and ensuring safe, evidence-based management of patellar instability in skeletally immature patients.

### 7.1. Lack of Pediatric-Specific Outcome Measures

Most studies evaluating outcomes after MPFL reconstruction or trochleoplasty utilize adult-derived patient-reported outcome measures (PROMs), such as the Kujala or Lysholm scores. While these tools assess symptoms and functional limitations, they may not fully capture the unique activity demands, growth-related concerns, or psychosocial factors relevant to children and adolescents. There is a need for validated pediatric-specific PROMs that can assess return-to-play, developmental milestones, and long-term joint health following orthopedic interventions, such as patellofemoral surgery [113,114,115]. Thus, incorporating such tools would improve postoperative tracking and allow for more meaningful comparisons across age groups and arthroscopic treatment modalities.

### 7.2. Need for Long-Term Studies on Skeletally Immature Patients

The long-term impact of MPFL reconstruction, particularly when performed using arthroscopic physeal-sparing techniques, remains relatively understudied. While short-term outcomes have shown low re-dislocation rates and improved function [69,74,92,93], there is limited data on physeal growth patterns, durability of graft integrity, and the development of patellofemoral osteoarthritis over time. Most pediatric studies involve fewer than 5 years of follow-up and focus on recurrence rates, leaving questions about cartilage preservation, growth disturbance, and patient-reported function unanswered. Longitudinal, prospective cohort studies are necessary to establish the safety and efficacy of these procedures, particularly those using arthroscopic techniques designed to minimize physeal injury.

### 7.3. Standardization of Indications and Rehabilitation Protocols

Variation in clinical practice is common, both in decision to operate and in postoperative management. In the literature, inconsistencies in the threshold for surgical intervention, especially following first-time dislocations in skeletally immature patients have been documented [116]. Anatomical risk factors such as trochlear dysplasia, patella alta, and TT-TG distance are considered but no universally accepted scoring system or decision workflow exists to guide when to proceed with surgery in children. Arthroscopy has become increasingly utilized not only as a therapeutic modality but also as a diagnostic adjunct in equivocal or recurrent cases, enabling real-time assessment of patellofemoral tracking, MPFL integrity under physiological loading conditions, and chondral damage. This capability may support earlier identification of intraarticular pathology not captured on static MRI, thereby influencing operation timing and surgical technique selection.

However, arthroscopic indications themselves remain non-standardized. In some clinics, diagnostic arthroscopy is routinely performed to evaluate for loose bodies and cartilage lesions even after first-time subluxation, while in others, it is reserved for patients with chronic instability and mechanical symptoms or may not used at all. This lack of consensus impacts clinical comparability and research reproducibility.

Likewise, rehabilitation protocols following arthroscopic MPFL reconstruction, lateral release, or trochleoplasty vary widely across institutions. Differences exist in initial weight-bearing status, the use of bracing, timing of range-of-motion exercises, and return-to-sports timelines. Pediatric patients, who differ from adults in healing capacity, compliance, and skeletal maturity, may benefit from arthroscopy-specific, age-adjusted rehabilitation protocols. This highlights the need for consensus-driven, pediatric-specific guidelines to optimize functional recovery and reduce the risk of recurrence or over-restriction during key growth periods [117]. Standardizing arthroscopic indications, procedural approaches, and rehabilitation pathways will be essential to improve outcomes and ensure consistency in the care of skeletally immature patients with patellar instability.

## 8. Clinical Considerations and Challenges

Managing patellar instability in skeletally immature patients presents several unique clinical challenges that require careful consideration to determine the best procedure for each individual patient. There are three large categories contributing to instability: traumatic injury, mechanical malalignment, and hypermobility syndromes. It is critical to understand the source of instability for each patient. In general, patients who sustain a traumatic injury will report a history of a sports related contact injury or higher energy injury such as a fall from a bicycle. Patients with hyperligamentous laxity syndromes can be detected by a history of other joint instabilities as well as on physical exams, including an assessment of Beighton’s Criteria for ligamentous laxity. It is also important to recognize that any given patient with patellar instability can have sources of instability relevant to two or three of these general classifications. As an example, a 14-year-old girl with hyperligamentous laxity may also have pathology related to mechanical malalignment. Furthermore, it is important to recognize that some patients with patellar instability may have malalignment in more than one plane. It is not uncommon for adolescents with a condition known as “Miserable Malalignment” to have femoral anteversion (malalignment in the axial plane), valgus knee alignment (malalignment in the coronal plane), and pronated feet (this contributes to malalignment in both the coronal and axial planes). Any physical therapy program or surgical plan for treatment must consider all of these factors in order to maximize the potential for success.

## 9. Rehabilitation

Post-operative rehabilitation following patellar instability reconstruction in skeletally immature patients should follow a structured, phase-based progression with criteria-driven milestones rather than arbitrary time points. Guidelines from leading pediatric institutions such as Massachusetts General Brigham outline a five-phase protocol [118,119] beginning with phase I (0–2 weeks) focusing on surgical protection, pain and swelling control, restoration of full active knee extension and gradual flexion to 60°, with partial weight-bearing and brace locked in extension during ambulation. Phase II (2–6 weeks) progresses to weight-bearing as tolerated, unlocking the brace as quadriceps control permits, achieving knee flexion towards 110–120°, and introducing close-chain strengthening and proprioceptive exercises [118].

Beyond six weeks, criteria-based progression continues, emphasizing limb symmetry in quadriceps strength (≥80% via isokinetic testing) and neuromuscular control assessed via single-leg squat mechanics, before advancing to running and agility drills [104,118,119,120]. Return-to-sports is typically targeted at around 10 months post-op [121], contingent on passing objective functional tests including hop testing (leg symmetry index ≥ 90%), strength symmetry, and absence of pain or effusion.

However, a recent work [121] highlights a critical limitation of hop-based testing. The leg symmetry index may be inappropriate for patients with patellar instability because bilateral deficits in strength and function can normalize the index and mask residual impairments [121]. This suggests that while hop testing remains useful, clinicians should supplement limb symmetry index with absolute performance measures, side-by-side comparisons of mechanics, and validated pediatric-specific PROMs. Notably, Saper et al. [122] reported that only 32% of adolescents achieve satisfactory hop test results at eight months, further underscoring the heterogeneity of recovery and the importance of individualized, multimodal clearance criteria. Together, these findings support a cautious, criteria-driven approach to return to sport that integrates objective strength and functional testing, highlighting the need for PROMs tailored to pediatric populations.

## 10. Strengths and Limitations

This review provides a comprehensive and clinically relevant synthesis of contemporary approaches to patellar instability in skeletally immature patients. By incorporating innovative techniques such as robotic-assisted navigation, 3D modeling, and bioengineered scaffolds, this review highlights cutting-edge strategies that may soon influence practice. The focus on pediatric-specific considerations, including physeal-sparing MPFL reconstruction, distinguishes this work from prior general reviews. Furthermore, the detailed historical framing of the Dejour classification and *menu à la carte* provides valuable context for understanding the evolution of surgical decision-making.

However, limitations of this paper should be acknowledged. First, the review is narrative in design, without quantitative meta-analysis, which may introduce selection bias. Second, the available literature is heterogeneous, spanning retrospective series, case reports, and small single-center studies, limiting the generalizability of conclusions. Third, there is a paucity of long-term follow-up, with most studies reporting less than 5 years of outcomes, leaving growth effects and osteoarthritis risk incompletely understood. Fourth, rehabilitation protocols remain variable across institutions, with limited evidence to guide standardized pediatric-specific pathways. Finally, there is a lack of validated pediatric outcome measures, which hampers cross-study comparisons and high-quality evidence synthesis.

## 11. Conclusions

Arthroscopy has become an essential tool in the diagnosis and management of patellar instability in pediatric and adolescent patients. It enables minimally invasive evaluation of patellofemoral anatomy, identification of chondral lesions, and treatment of intraarticular pathology, such as loose bodies. Arthroscopic techniques also facilitate reconstruction procedures, such as MPFL reconstruction, with improved visualization and precision. When appropriately applied, these procedures can significantly improve knee stability, restore function, and reduce recurrence rates in skeletally immature patients.

Management strategies for patellar instability must be tailored to the unique anatomical and developmental characteristics of each patient. Individualized, growth-aware approaches, such as physeal-sparing techniques, growth modulation procedures, and patient-specific surgical planning, are critical to preserving joint integrity and preventing long-term complications. Preoperative planning using 3D modeling, and advances in biologics and cartilage restoration, further support safe and effective care.

Despite these advancements, important challenges remain. The field would benefit from the development of pediatric-specific outcome measures, standardization of surgical indications, and long-term studies to assess durability and safety. Collaborative, interdisciplinary research teams are essential to address these knowledge gaps. Innovation in imaging, surgical instrumentation, and biologic augmentation should be integrated with clinical expertise to advance care for young patients with patellar instability.

Future studies should prioritize the following: (1) validation of pediatric-specific PROMs capable of capturing activity demands, growth concerns, and psychological impact; (2) multicenter prospective trials evaluating the safety and efficacy of physeal-sparing MPFL techniques; (3) development of international registries to pool large datasets and enable cross-population analyses; (4) cost-effectiveness studies comparing emerging technologies such as robotics and 3D planning to standard approaches; and (5) long-term follow-up (≥10 years) assessing growth plate effects, graft durability, and the risk of secondary patellofemoral osteoarthritis. Together these efforts will create the evidence base necessary to translate current innovation into sustainable, evidence-driven pediatric orthopedic care.

Arthroscopy offers a powerful platform for both current treatment and future innovation. With continued refinement to surgical techniques and research protocols, the field is well positioned to improve the quality, safety, and personalization of care for pediatric and adolescent patients with patellofemoral instability.

## Figures and Tables

**Table 1:** Diagnostic Classification Systems and Thresholds for Patellar Instability. Italicized term is a French phrase that has been incorporated into English usage but is still treated as a loan expression.

Classification/ Measure	Risk Factor	Method	Threshold for Abnormality	Notes	Limitations
Caton–Deschamps Index [36]	Patella alta	Ratio of distance from inferior patellar pole to tibial plateau divided by the length of the patellar articular surface on lateral radiograph	Greater than 1.2 indicates patella alta	Preferred in pediatric populations due to growth plate visibility Sensitive to knee flexion angle at the time of imaging; does not account for trochlear morphology or dynamic engagement	
Insall–Salvati Ratio [38]	Patella alta	Ratio of patellar tendon length to patellar length	Greater than 1.2 indicates patella alta	Common in adults, but less reliable in immature knees. Some success in skeletally immature populations [38] Inconsistent reproducibility in pediatric knees	
TT-TG Distance [25]	Lateralization to tibial tuberacle	Measured on axial MRI or computer tomography (CT) as horizontal distance between tibial tubercle and trochlear groove	Greater than 20 mm considered abnormal	Greater than 20 mm associated with instability; 15–20 mm may be borderline and context-dependent in pediatric cases [39] Values vary with imaging modality (MRI vs. CT) and knee flexion angle	
Dejour Classification (2025) [40]	Trochlear dysplasia	Integrated assessment using lateral radiographs and MRI; emphasizes functional evaluation of patellofemoral tracking	Redefined dysplasia categories with emphasis on dynamic morphology; linked directly to updated *menu à la carte* treatment framework.	Supersedes earlier A-D system; provides improved risk stratification and clearer surgical indications (trochleoplasty reserves for select high-grade dysplasia, not skeletally immature patients) [40]	Requires advanced imaging; some parameters prone to inter-observer variability
TT-PCL [26,27,28,29]	Rotational alignment, lateralization of the tibial tubercle	Measured on axial MRI or CT as the distance from the tibial tubercle to the posterior cruciate ligament insertion	Greater than 20 mm is typically considered abnormal [29]	Used alongside TT-TG distance to evaluate rotational malalignment and refine surgical planning, especially important in skeletally immature patients	Normative pediatric values not well established

Italicized term (*menu à la carte*) is a French phrase that has been incorporated into English usage but is still treated as a loan expression.

**Table 2:** Updated Dejour [40] MRI Parameters for Patellofemoral Instability.

Parameter	Definition	Imaging Plane	Threshold	Clinical Significance
Spur height	Bony supratrochlear spur height	Sagittal	>5 mm	Indicates high-grade trochlear dysplasia
Cranial trochlear orientation	Orientation of proximal trochlea vs. posterior condyles	Axial	Positive value	Reflects cranial malorientation; predicts patellar maltracking
Trochlear groove height	Angle between trochlear groove and posterior condylar axis	Coronal	>11°	Excessive obliquity indicates severe dysplasia
Patellar height index	Ratio of patellar length to articular contact length	Sagittal	>1.16	Identifies patella alta, risk of delayed trochlear engagement
Sagittal patellofemoral engagement	Ratio of engaged patella length to total patellar length	Sagittal	<0.38	Low value = poor engagement, instability risk
TT-TG distance	Horizontal distance tibial tubercle to trochlear groove	Axial	≥14 mm	Pathologic lateralization, informs consideration for TTO

**Table 3:** Comparative Fixation Strategies for Physeal-Sparing MPFL Reconstruction in Skeletally Immature Patients.

Site	Fixation	Indications	Advantages	Limitations/Risks	Ease of Use	Evidence
Femur	Short epiphyseal socket + interface screws	Epiphyseal anatomy permits short tunnel distal to physis	Anatomic fixation; firm stability	Risk of physeal breach; hardware in small epiphysis Moderate Level IV		
Femur	Short socket + cortical button/adjustable loop	Need for fine tension control; physeal respect	Micro-adjustable tension; preserves epiphysis	Requires cortical bridge; hardware prominence Moderate Level IV [13,95]		
Femur	Adductor sling (soft tissue loop)	Younger patients; desire to avoid drilling	No bone tunnels; fully physeal sparing	Creep/lengthening; soft tissue isometry dependent Moderate Level IV [69,70,71,72]		
Femur	Epiphyseal suture anchors	Tunnel-free physeal sparing	Minimal bone removal	Pull-out risk; limited long-tern outcomes	Easy-Moderate	Level III–IV [95,96]
Patella	Medial anchors (2 low profile)	Most pediatric cases	Minimizes fracture risk; small sockets	Anchor pull-out; cost	Easy-Moderate	Level III–IV [78,79,97,98]
Patella	Protected unicortical tunnels	When anchors unavailable	Strong fixation; inexpensive	Risk of fracture if bicortical or oversized	Moderate	Level IV [98,99]
Patella	Full-thickness transverse tunnels	Historically used	High stiffness	High fracture risk in pediatrics	Moderate	Consensus to avoid [80]

**Table 4:** Comparison of Patellar Instability Procedures by Skeletal Maturity.

Technique	Indication	Skeletally Immature	Advantages	Limitations	Level of Evidence
Physeal-sparing MPFL	Recurrent instability, open physes	Preferred	Preserves growth plates; good outcomes	Technical complexity; limited long-term data	Level III–IV [43,65,66,67,68]
Adductor sling	Younger children, avoid tunnels	Yes	No bone drilling; physeal safe	Graft creep risk	Level IV [69,70,71,72]
Epiphyseal socket MPFL	Older adolescents; nearing maturity	Selective	Anatomic fixation Risk of epiphyseal breach Level IV [13,69,70]		
TTO	TT-TG > 20 mm, patella alta	Contraindicated	Corrects alignment	Growth disturbance risk	Level III [44,49,100,101]
Trochleoplasty	Severe trochlear dysplasia	Contraindicated	Corrects bony dysplasia	Contraindicated in open physes	Level III [102,103,104,105,106,107]
Soft tissue procedures	Adjunctive instability	Yes	Minimally invasive	Variable outcomes	Level IV [14,61,72,74,75]

**Table 5:** Technology Readiness and Evidence Quality for Innovations in Pediatric Patellar Instability.

Innovation	Evidence Strength	Clinical Availability	Cost	Learning Curve
Robot-assisted navigation [18]	Small single-center retrospective studies (*n* = 20–40; <2 years follow up)	Available in tertiary centers only	High capital/equipment cost	Requires specialized training
Bioengineers Scaffolds [108]	Mostly preclinical and animal models	Experimental, not widely available	Variable, currently high	Straightforward arthroscopic integration if validated
3D Modeling and Patient-Specific Planning [53,109,110,111,112]	Small retrospective series (*n* < 50); promising feasibility	Growing availability at academic centers	Moderate (software)	Moderate learning curve, aids planning

## Data Availability

Not applicable.

## Abbreviations

The following abbreviations are used in this manuscript:

TT-TG	Tibial tuberosity to trochlear groove
TT-PCL	Tibial tuberosity to posterior cruciate ligament
MPFL	Medial patellofemoral ligament
3D	Three-dimensional
MRI	Magnetic resonance imaging
CT	Computed tomography
TTO	Tibial tubercle osteotomy
2D	Two-dimensional
PROMs	Patient-reported outcome measures
AP	Anteroposterior
